# A regulatory circuitry comprising TP53, *miR-29* family, and SETDB1 in non-small cell lung cancer

**DOI:** 10.1042/BSR20180678

**Published:** 2018-09-14

**Authors:** Bifeng Chen, Jingdong Wang, Jieling Wang, Huan Wang, Xiuli Gu, Liang Tang, Xianhong Feng

**Affiliations:** 1 Department of Biological Science and Technology, School of Chemistry, Chemical Engineering and Life Sciences, Wuhan University of Technology, Wuhan, China; 2 Center of Reproductive Medicine, Tongji Medical College, Huazhong University of Science and Technology; 3 Department of Reproductive Genetics, Wuhan Tongji Reproductive Medicine Hospital, Wuhan, China; 4 Department of Human Anatomy, Histology and Embryology, Institute of Neuroscience, Changsha Medical University, Changsha, China; 5 Clinical Laboratory, Wuhan Xinzhou District People’s Hospital, Wuhan, China

**Keywords:** H3K9 methylation, miR-29s, non-small cell lung cancer, SETDB1, TP53

## Abstract

Lung cancer is a malignant tumor with high fatality rate and causes great harm to human economic life. Non-small cell lung cancer (NSCLC) is the most common type of lung cancer. With the rapid development of epigenetic study in the last decade, the understanding of the pathogenesis of lung cancer and the development of personalized treatment of lung cancer are picking up pace. Previous studies showed that *miR-29* family members (*miR-29s*; *miR-29a, -29b*, and *-29c*) are down-regulated in most human cancers, including NSCLC, but their biological roles in tumorigenesis and their regulation mechanism are still not fully elucidated. Herein, we reported that the *miR-29a, -29b* and, *-29c* were coincidently down-regulated in NSCLC, and the histone H3K9 methyltransferase SET domain, bifurcated 1 (SETDB1) was directly targetted by *miR-29s*. Moreover, SETDB1 negatively regulated the expression of TP53 and overexpression of SETDB1 down-regulating the expression of *miR-29s*, while TP53 positively regulated the expression of *miR-29s* and overexpression of TP53 down-regulated the expression of SETDB1. On the other side, as a downstream target of TP53, the H3K9 methyltransferase Suv39h1 was also down-regulated by *miR-29s* via up-regulating TP53 expression. The further detection of H3K9 methylation status after changes in *miR-29s* expression revealed that they negatively regulated the levels of H3K9 di- and trimethylation in NSCLC. Collectively, our findings highlight a TP53/*miR-29s*/SETDB1 regulatory circuitry and assign a role of H3K9 methylation regulator to *miR-29s*, which may be a potential therapeutic target in the treatment of NSCLC.

## Introduction

Lung cancer is the most common cancer worldwide, accounting for 1.3 million deaths annually [1]. Non-small cell lung cancer (NSCLC) represents approximately 80% of lung cancers, which includes adenocarcinoma, squamous-cell carcinoma, and large-cell carcinoma [2]. Currently, the treatment options for inoperable lung cancer patients are very limited [3]. In this regard, new biomarkers for early diagnosis and new targets for therapy are eagerly awaited to improve the survival rate of lung cancer patients.

As an important epigenetic modification, histone methylation plays a crucial role in regulating gene expression in normal mammalian development, and deregulation of histone modification is a common hallmark of human cancers [4]. Generally, five lysines on histone H3 (Lys^4^, Lys^9^, Lys^27^, Lys^36^, and Lys^79^) and one lysine on histone H4 (Lys^20^) can undergo methylation by the histone methyltransferases (HMTases), and each of these lysine residues can be mono-, di-, and trimethylated [5,6]. *In vivo*, H3K9 methylation is specifically catalyzed by SUV39H1/2 (suppressor of variegation 3-9 homolog 1), SET domain, bifurcated 1 (SETDB1), and G9a [7]. Importantly, previous studies demonstrated that SUV39H1 [8,9] and SETDB1 [10,11] are highly expressed in lung cancer, and the dysregulation of SUV39H1 and SETDB1 accelerates the development of lung cancer [9,11], suggesting that aberrant H3K9 methylation plays a critical role in lung carcinogenesis.

MiRNAs are a class of endogenous small non-coding regulatory RNAs of approximately 22 nts [12], that are able to bind to specific sites typically present in the 3′-UTRs of their target genes and mediate either mRNA decay with perfect base pairing or translational blockade with imperfect base pairing [13]. A growing number of evidences have shown that miRNAs have key roles in the regulation of cellular processes and that their dysregulation is essential to keep the malignant phenotype of cancer cells [14]. The *miR-29* family (*miR-29s*), containing three members (*miR-29a, -29b*, and *-29c*), has been shown to be down-regulated in lung cancer [15], especially in NSCLC [16,17]. However, the molecular biology underlying the down-regulation of *miR-29s* in NSCLC remains largely unknown.

Recently, Wong et al. [18] demonstrated that *miR-29a* modulated SETDB1 expression by directly binding to 3′-UTR region of SETDB1 in liver cancer. Since the sequences of mature *miR-29* family members are highly homologous and contain the same ‘seed sequence’, it was suspected that the whole *miR-29* family could directly target SETDB1 in NSCLC. Moreover, previous studies reported that SETDB1 negatively regulates the expression of TP53 [11], and TP53 positively regulates the transcription of *miR-29s* [19]. These facts together appear to suggest that TP53, *miR-29s*, and SETDB1 may form a regulatory circuitry in NSCLC. On the other side, SUV39H1 is a repression target of TP53 [6], thus *miR-29s* would down-regulate Suv39h1 expression by activating TP53. Therefore, it was reasonable to speculate that *miR-29s* could regulate the H3K9 methylation status by targetting directly SETDB1 and indirectly Suv39h1 in NSCLC.

## Materials and methods

### Primary NSCLC specimens

Thirty primary NSCLC samples (grades 3 and 4) and their paired adjacent normal samples were obtained from NSCLC patients who had undergone surgical resection at the Wuhan Xinzhou District People’s Hospital, with consent obtained from patients. Further, no deleterious TP53 mutations were detected in all the included NSCLC samples. The present study was approved by the Ethical Committees of Wuhan University of Technology.

### Cell culture and transfection

NSCLC cell lines (A549 and PC14) were purchased from (ATCC Manassas, VA, U.S.A.) and were grown in RPMI 1640 medium plus 10% FBS in a 37°C humidified incubator supplied with 5% CO_2_. Human bronchial epithelial (BEAS-2B) cells (ATCC, Rockville, MD) were cultured in DMEM supplemented with 10% FBS, 2 mM l-glutamine, and 5% penicillin/streptomycin at 37°C in a humidified atmosphere with 5% CO_2_.

Synthetic double-stranded *miR-29a, -29b*, and *29c* mimics, scramble oligonucleotides used as negative control (Scr NC) (GenePharma, Shanghai, China) at a final concentration of 20 nM were introduced into A549, PC14, or BEAS-2B cells by Lipofectamine 2000 kit (Invitrogen, Carlsbad, CA) according to the manufacturer’s instructions. Transfected cells were harvested at 48 h.

### Modulation of SETDB1 and TP53 expression

The siRNAs specifically targetting SETDB1 (siSETDB1) and TP53 (siTP53) were obtained commercially (GenePharma, Shanghai, China). The synthetic siSETDB1, siTP53, or Scr NC at a final concentration of 20 nM were introduced into A549, PC14, or BEAS-2B cells using Lipofectamine 2000 kit (Invitrogen, Carlsbad, CA) as previously described by cell culture and transfection methods. Wild-type TP53 expression vector (pEGFP-TP53, plasmid 12091, GeneBank ID: AAD28628.1) was bought from Addgene. The SETDB1 ORF was amplified by PCR from human cDNA and inserted into the pcDNA3.1(+) to construct the pcDNA3.1-SETDB1 expression vector. The SETDB1 cloning primers were 5′-GGGGTACCACAAAAGCATGTCTT-3′ (forward) and 5′-AGGCTCTAGACTAAAGAAGACGTCC-3′ (reverse). The vector backbone pEGFP-N1 and pcDNA3.1(+), used as control vector, was purchased from Clontech Laboratories, Inc. (Mountain View, CA, U.S.A.). Transfected cells were harvested at 48 h.

### Construction of luciferase reporter vector and dual luciferase reporter assay

For *miR-29s* target validation, wild-type (WT) and mutated (Mut) *miR-29s* binding sequences in the SETDB1 3′-UTR were amplified by PCR from genomic DNA and inserted into the firefly/*Renilla* dual reporter vector pmirGLO (Promega, Madison, WI) at the 3′-end of the firefly luciferase gene. The primers for construction of luciferase reporter vector were as follows: pmirGLO-SETDB1-3′UTR-WT, forward 5′- CTAGCTAGCGAAGGTTACACCACGATCG-3′ and reverse 5′-GCTCTAGAATCTATGTGTCCGAGGTAA-3′; pmirGLO-SETDB1-3′UTR-Mut, forward 5′-CTAGCTAGCGAAGGTTACACCAGTCACG-3′ and reverse 5′-GCTCTAGAATCTATGTGTCCGAGGTAA-3′. A total of 3×10^4^ 293T cells were plated in each well of a 24-well plate 1 day before transfection. 293T cells were co-transfected with pmirGLO-SETDB1-3′UTR-WT construct or pmirGLO-SETDB1-3′UTR-Mut construct and *miR-29a, 29b, 29c* mimics or Scr NC or co-transfected with empty pmirGLO plasmid and *miR-29a, 29b, 29c* mimics or Scr NC by Lipofectamine 2000 (Invitrogen, Carlsbad, CA) as previously described by cell culture and transfection methods. Four wells for each group in a single independent experiment. A luciferase activity assay was performed 48 h after transfection with the dual luciferase reporter assay system (Promega). The relative luciferase activity was normalized with *Renilla* luciferase activity.

### Reverse-transcription reaction and quantitative real-time PCR

The expression levels of genes were quantitated with RT-qPCR and calculated with the 2^−ΔΔ*C*^_t_ method [20]. The harvested cells were placed in TRIzol® Reagent (Invitrogen, Carlsbad, CA), and total RNAs were extracted according to the TRIzol manufacturer’s instructions. Total RNA (1 μg) was primed by random hexamers and converted into cDNA using SuperScript III (Invitrogen). The SYBR Green-based real-time PCR was performed in an Applied Biosystems 7900HT Fast Real-Time PCR System (Applied Biosystems, Foster City, CA, U.S.A.), and GAPDH was used as an endogenous control to normalize the amount of total mRNA in each sample. The primer sequences for quantitative real-time PCR (qPCR) analysis were as follows: SETDB1, forward 5′-GGGCAAGGGTGTTTTCATTAAC-3′ and reverse 5′-GTTAGTTGATGGCAGGCACACTT-3′; TP53, forward 5′-TCCACTACAACTACATGTGTAAC-3′ and reverse 5′-GTGAAATATTCTCCATCCAGTG-3′; SUV39H1, forward 5′-ATATCCAGACTCAGAGAGCACC-3′ and reverse 5′-CAGCTCCCTTTCTAAGTCCTTG-3′; GAPDH, forward 5′-TGCACCACCAACTGCTTAGC-3′ and reverse 5′-GGCATGGACTGTGGTCATGAG-3′. The expression of mature *miR-29a, -29b*, and *29c* were measured using the well-established stem-loop RT-qPCR method as previously described [21], and the expression level of *miR-29s* were normalized by U6 RNA. Real-time PCR was performed with a standard SYBR-Green PCR kit protocol on ABI 7900HT Fast Real Time PCR system (Applied Biosystems, Foster City, CA). The RT primers for *miR-29s* and U6 were as follows: *miR-29a*, 5′-GTCGTATCCAGTGCAGGGTCCGAGGTATTCGCACTGGATACGACTAACCG-3′; *miR-29b*, 5′-GTCGTATCCAGTGCAGGGTCCGAGGTATTCGCACTGGATACGACAACACTG-3′; *miR-29c*, 5′-GTCGTATCCAGTGCAGGGTCCGAGGTATTCGCACTGGATACGACTAACCG-3′; U6, 5′-AAAATATGGAACGCTTCACGAATTTG-3′; The primer sequences for qPCR analysis were as follows: *miR-29a*, forward 5′-TGCGCTAGCACATCTGAAAT-3′; *miR-29b*, forward 5′-CTGGAGTAGCACCATTTGAAAT-3′; *miR-29c*, forward 5′-CTGGAGTAGCACCATTTGAAAT-3′; *miR-29s*, reverse 5′-GTGCAGGGTCCGAGGT-3′; U6, forward 5′-CTCGCTTCGGCAGCACATATACT-3′ and reverse 5′-ACGCTTCACGAATTTGCGTGTC-3′.

### Western blot analysis

Whole cell lysates were prepared in 1× SDS buffer. Proteins in the same amount were separated by SDS/PAGE and transferred on to PVDF membranes. After incubation with antibodies specific for either SETDB1 (HPA018142, Sigma–Aldrich), TP53 (SC-126, Santa Cruz Biotechnology), β-Actin (A1978, Sigma–Aldrich), Histone H3 (ab1791, Abcam), H3K9me2 (ab1220, Abcam), and Lys^9^ residue on histone H3 (H3K9me3) (ab8898, Abcam), the blots were incubated with goat anti-mouse (ab6789, Abcam) or goat anti-rabbit (ab6721, Abcam) and visualized with ECL.

### Statistical analysis

The differences for luciferase assay and RT-qPCR data were determined by two-tailed Student’s *t*test, data were expressed as means and S.D. from at least three independent experiments. All *P*-values were two-sided and were obtained with the SPSS 16.0 software package (SPSS, Chicago, IL). A *P*-value <0.05 was considered statistically significant (*, *P*<0.05; **, *P*<0.01; ***, *P*<0.001).

## Results

### Expression levels of all the *miR-29* family members are markedly down-regulated in NSCLC

**Figure 1:**
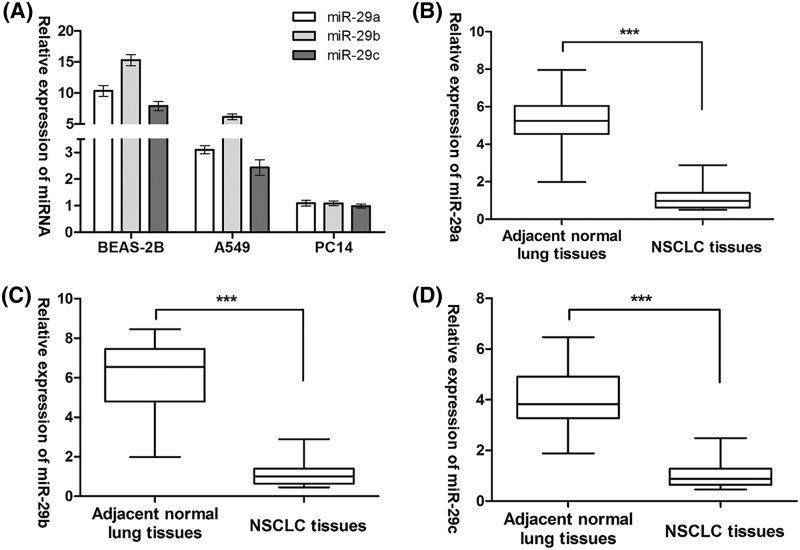
Expression of all the *miR-29* family members is markedly down-regulated in NSCLC (**A**) The expression of *miR-29* family members was assessed by RT-PCR in BEAS-2B, A549, and PC14 cells. (**B**) *miR-29a* mRNA expression, (**C**) *miR-29b* mRNA expression, and (**D**) *miR-29c* mRNA expression in primary NSCLC samples and their paired adjacent normal samples. ***P<0.001

We first performed Taqman stem-loop RT-PCR in two NSCLC cell lines (A549 and PC14) and a human bronchial epithelial cell line (BEAS-2B) to analyze the expression patterns of *miR-29s*. The results indicated a similar tendency of expression change, that is, a significantly decreased expression levels of *miR-29a, -29b*, and *-29c* were detected in NSCLC cell lines as compared with BEAS-2B cells (Figure 1A). Concordantly, the same observation was found in the comparison of *miR-29a, -29b*, and *-29c* expression between 30 primary NSCLC samples and their paired adjacent normal samples (Figure 1B–D). These data suggested that *miR-29s* are markedly down-regulated in NSCLC.

### SETDB1 is the common target of the *miR-29* family members

**Figure 2:**
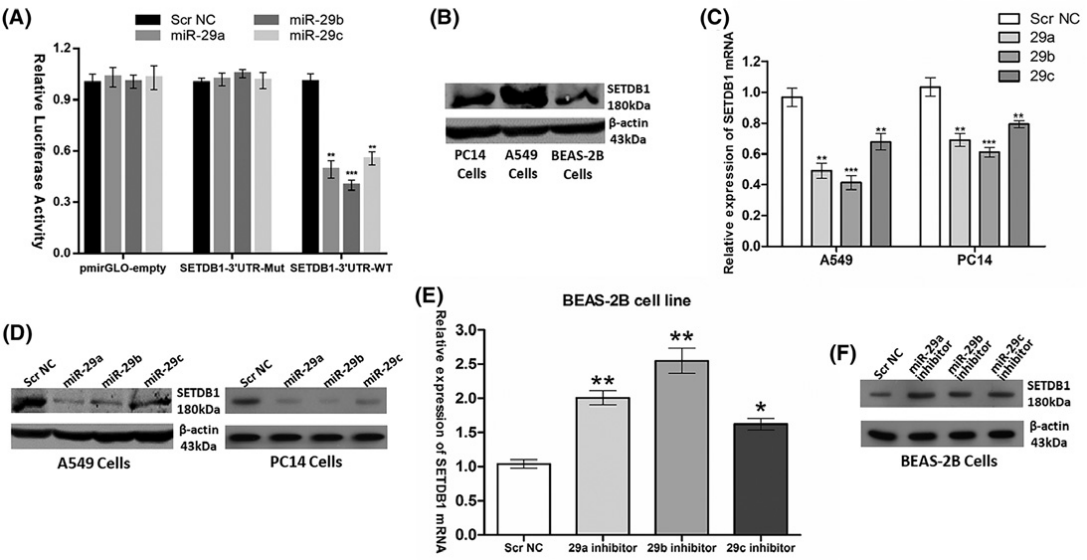
SETDB1 is the common target of the *miR-29* family members (**A**) Dual luciferase assay of 293T cells co-transfected with the luciferase constructs containing the SETDB1 3′-UTR_WT or SETDB1 3′-UTR_Mut as well as *miR-29s* mimics or Scr NC. (**B**) SETDB1 protein levels was assessed in PC14, A549, and BEAS-2B cells. (**C**) *SETDB1* mRNA levels and (**D**) SETDB1 protein levels after transfection of *miR-29s* mimics or Scr NC into A549 and PC14 cells. (**E**) *SETDB1* mRNA levels and (**F**) SETDB1 protein levels after transfection of *miR-29s* inhibitors or Scr NC into BEAS-2B cells. *P<0.05; **P<0.01; ***P<0.001

A previous *in vitro* luciferase assay showed that overexpression of *miR-29a* significantly repressed the luciferase activity of wild-type SETDB1 3′-UTR tagged reporter. Consistently, *miR-29a* also negatively regulated the *SETDB1* mRNA and protein levels in liver cancer [18]. To confirm and investigate the negative regulator role of the whole *miR-29* family on SETDB1, we first constructed a luciferase report system containing wild-type and mutated *miR-29s* binding sequences of SETDB1 3′-UTR. As shown in Figure 2A, when co-transfected with *miR-29a, -29b*, or *-29c* mimics, the relative luciferase activities of pmirGLO-SETDB1-3UTR-WT luciferase reporter in 293T cells were significantly suppressed by at least 40% compared with the transfection with Scr NC, empty pmirGLO vector, or pmirGLO-SETDB1-3UTR-Mut vector. In contrast with the expression patterns of *miR-29s* in NSCLC cell lines (A549 and PC14) and BEAS-2B cells, elevated expression of SETDB1 protein levels was noted in NSCLC cell lines as compared with BEAS-2B cells (Figure 2B). Next, mRNA levels (as measured by RT-qPCR) and protein levels (as measured by Western blot analysis) of SETDB1 were measured 48 h after transient transfection of *miR-29s* mimics into A549 or PC14 cells. The results showed that *miR-29s* ectopic expression caused a significant decrease in SETDB1 expression at both mRNA and protein levels in comparison with Scr NC in A549 and PC14 cells (Figure 2C,D). Knocking down of *miR-29s* expression in BEAS-2B cells using an anti-miRNA inhibitor specific for *miR-29a, -29b*, or *-29c* resulted in significant up-regulation of *SETDB1* mRNA and protein levels (Figure 2E,F).

### TP53 represses the expression of SETDB1 via increasing *miR-29s* expression

**Figure 3:**
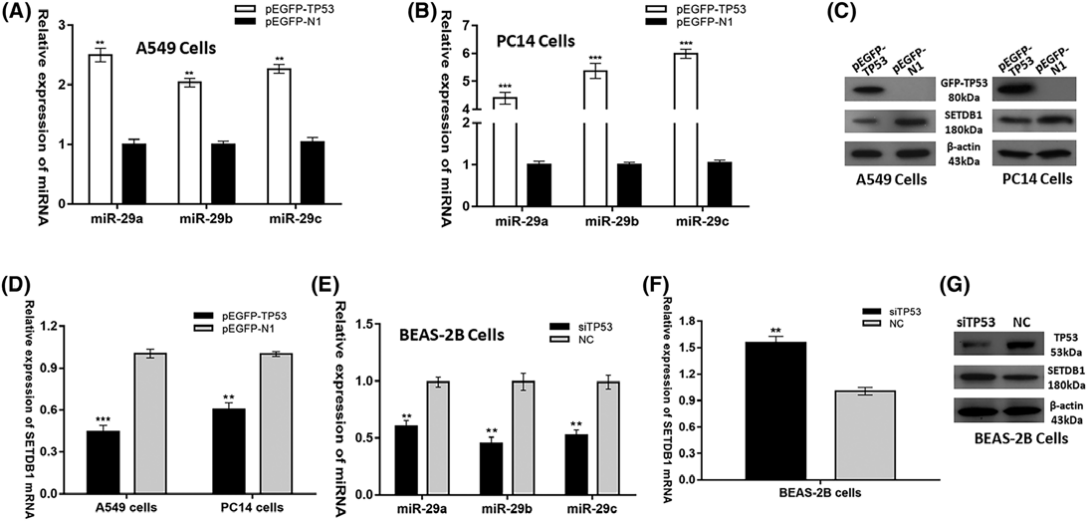
TP53 represses the expression of SETDB1 via increasing *miR-29s* expression *miR-29s* mRNA expression after transfection of pEGFP-TP53 vector or pEGFP-N1 control vector into (**A**) A549 cells and (**B**) PC14 cells. (**C**) SETDB1 protein level and (**D**) *SETDB1* mRNA expression after transfection of pEGFP-TP53 vector or pEGFP-N1 control vector into A549 and PC14 cells. (**E**) *miR-29s* mRNA expression, (**F**) *SETDB1* mRNA expression and (**G**) SETDB1 protein level after transfection of siTP53 or Scr NC into BEAS-2B cells. **P<0.01; ***P<0.001

Previous knowledge suggested that TP53 is responsible for the transcriptional activation of *miR-29s*. Luciferase assays revealed that TP53 was able to activate promoter activity of the hsa-*miR-29b*-1B29a (*miR-29a* and *29b*) and hsa-*miR-29b*-2B29c (*miR-29b* and *29c*), and ectopically expressed TP53 could induce the expression of mature *miR-29a, 29b*, and *29c* [19]. Moreover, *miR-29s* have been validated to directly target SETDB1. Therefore, it was conceivable to propose that TP53 represses SETDB1 expression via increasing *miR-29s* expression. To validate our hypothesis, we tested the effects of TP53 changes on *miR-29s* and SETDB1 expression. Indeed, forced expression of TP53 in the two NSCLC cell lines (A549 and PC14) significantly increased the levels of *miR-29s* (Figure 3A,B) while inhibited the expression levels of *SETDB1* mRNA and protein (Figure 3C,D). Likewise, silencing TP53 in BEAS-2B cells reduced the levels of *miR-29s* (Figure 3E) while increased the expression levels of SETDB1 mRNA and protein (Figure 3F,G). Collectively, these results decisively proved that TP53 represses the expression of SETDB1 via increasing *miR-29s* expression in NSCLC.

### SETDB1 represses the expression of *miR-29s* via decreasing TP53 expression

**Figure 4:**
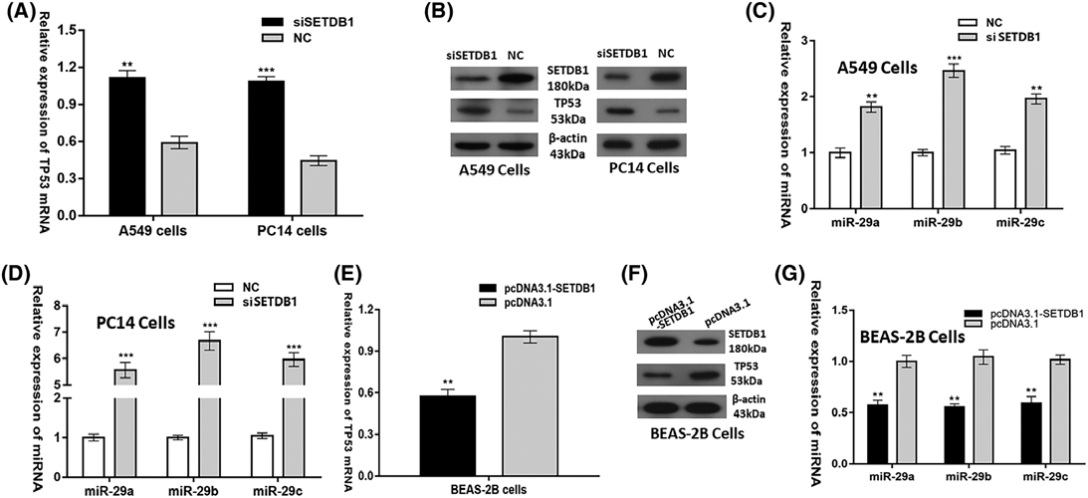
SETDB1 represses the expression of *miR-29s* via decreasing TP53 expression (**A**) *TP53* mRNA levels and (**B**) TP53 protein levels (**C**,**D**) *miR-29s* levels after transfection of siSETDB1 scrambled oligonucleotides as the negative control into A549 and PC14 cells. (**E**) *TP53* mRNA levels and (**F**) TP53 protein levels and (**G**) *miR-29s* levels after transfection of pcDNA3.1-SETDB1 vector or pcDNA3.1 control vector into BEAS-2B cells. **P<0.01; ***P<0.001

It was previously reported that SETDB1 is a negative regulator of tumor suppressor TP53 in NSCLC [11]. This negative regulation was conformed in our study as knocking down of SETDB1 in A549 and PC14 cells greatly up-regulated TP53 expression (Figure 4A,B), while overexpression of SETDB1 in BEAS-2B cells significantly down-regulated TP53 expression (Figure 4E,F). Since TP53 positively regulates the *miR-29s* expression, it was interesting to find that SETDB1 negatively regulated the expression of *miR-29s* in NSCLC (Figure 4C,D,G). Taken together, our data demonstrated that SETDB1 represses the expression of *miR-29s* via decreasing TP53 expression.

### The correlation of TP53, *miR-29s*, and SETDB1 expression levels in NSCLC

**Figure 5:**
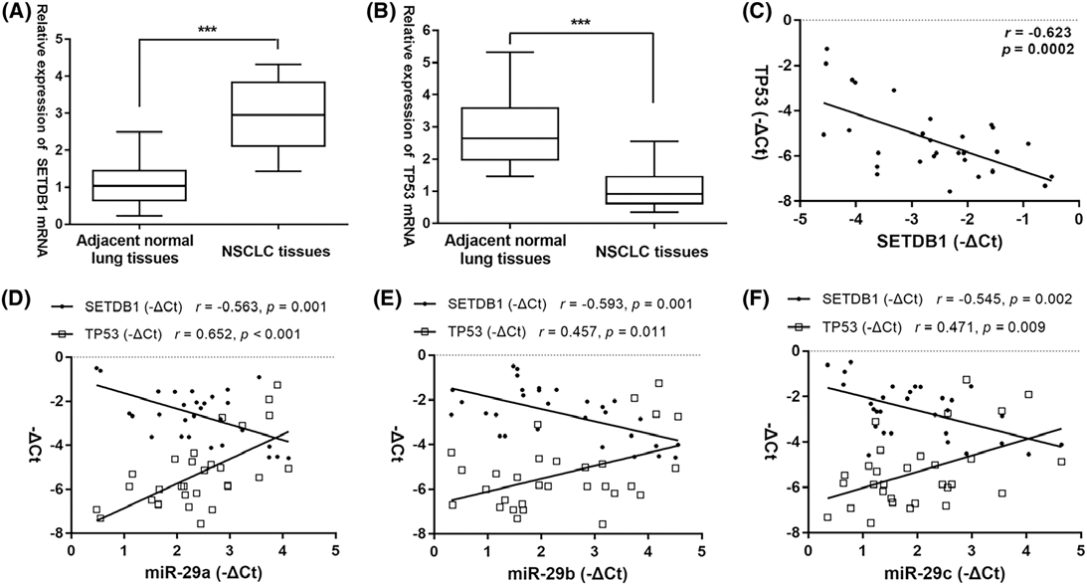
The correlation of TP53, *miR-29s*, and SETDB1 expression levels in NSCLC (**A**) *SETDB1* mRNA levels and (**B**) *TP53* mRNA levels in primary NSCLC samples and their paired adjacent normal samples. (**C**) *SETDB1* and *TP53* mRNA levels were inversely correlated in primary NSCLC samples. (**D**–**F**) Pearson correlation analysis between *miR-29s* and *SETDB1* mRNA levels, as well as between *miR-29s* and *TP53* mRNA levels in primary NSCLC samples. ***P<0.001

To further elucidate the relationship between *miR-29s* expression levels and TP53/SETDB1 expression levels in primary NSCLC samples, we first detected *TP53* and *SETDB1* mRNA expression in clinical tissues. Increased *SETDB1* mRNA levels and decreased *TP53* mRNA levels were observed in NSCLC tissues than in adjacent normal lung tissues (Figure 5A,B), and the Pearson correlation analysis further showed that their mRNA levels were negatively correlated in NSCLC samples (*n*=30, *r* = −0.623, *P*=0.0002. Figure 5C). Next, the scatter plot and the Pearson correlation analysis demonstrated that the expression levels of *miR-29a*, -*29b*, and *-29c* were negatively correlated with *SETDB1* mRNA levels but positively correlated with *TP53* mRNA levels, respectively (Figure 5D–F). These data suggested that the TP53, *miR-29s*, and SETDB1 appear to modulate the expression of each other and form a self-regulated circle loop in NSCLC.

### *MiR-29s* affect the H3K9 methylation status in NSCLC

**Figure 6:**
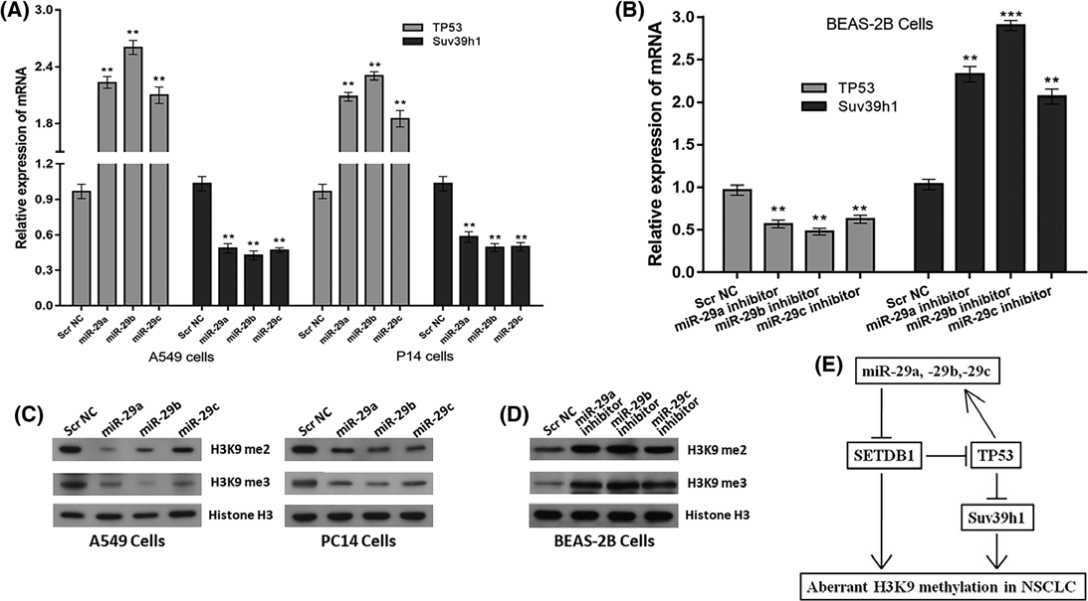
*MiR-29s* affect the H3K9 methylation status in NSCLC (**A**) *TP53* and *Suv39h1* mRNA levels after transfection of *miR-29s* mimics or Scr NC into A549 and PC14 cells. (**B**) *TP53* and *Suv39h1* mRNA levels after transfection of miR-29s inhibitors or Scr NC into BEAS-2B cells. Histone H3K9 methylation status in (**C**) NSCLC cells (A549 and PC14) with overexpressed *miR-29s* and (**D**) BEAS-2B cells with downexpressed *miR-29s* were examined by Western blot using antibodies specific for H3K9me2 and H3K9me3 (di- and trimethylation of H3K9, respectively); expression levels of histone H3 protein were used as an internal control. (**E**) Schematic illustration of the proposed TP53/*miR-29s*/SETDB1 regulatory circuitry in NSCLC. **P<0.01; ***P<0.001

Our newly identified *miR-29s*/SETDB1/TP53 regulatory circuitry indicated that *miR-29s* positively regulate TP53 expression via directly targetting SETDB1. Importantly, TP53 was previously reported to negatively regulate Suv39h1 expression and ectopically expressed TP53 abrogates the H3K9me3 heterochromatin mark that was added by Suv39h1 [6]. Thus, we here explored the potential interaction of *miR-29s* and Suv39h1. Interestingly, the modulation of *miR-29s* expression resulted in corresponding positive changes of TP53 expression and negative changes of Suv39h1 expression (Figure 6A,B). These observations suggested that *miR-29s* indirectly target Suv39h1 via TP53 in NSCLC. Considering that SETDB1 and Suv39h1 being as the histone H3K9 methyltransferases, it was reasoned that their regulator, *miR-29s*, would affect the H3K9 methylation status in NSCLC. Indeed, decreased H3K9 di- and trimethylation were noted in both A549 and PC14 cells when *miR-29a, -29b*, and -*29c* were overexpressed (Figure 6C), while elevated H3K9 di- and trimethylation was observed in BEAS-2B cells when *miR-29a, -29b*, and *-29c* were downexpressed (Figure 6D). Collectively, these data assigned a role of H3K9 methylation regulator to *miR-29s* in NSCLC.

## Discussion

Since the inception of epigenetics in the 1940s, discoveries implicating its role in cancer are mounting continuously [22]. As the important epigenetic regulators, histone methylation and miRNAs are widely studied. Dysregulation of histone methylation and aberrant miRNA profiling have been found as the hallmarks of cancer [4,14], but the mechanisms underlying these abnormities remain largely unknown.

As a master regulator, miRNA involves in multiple cellular processes during normal development and diseases [23]. Previous studies mainly focussed on the elucidation of miRNA functions [24]. Numerous studies collectively showed that *miR-29* family can be an effective regulator of tumorigenesis and cancer progression by targetting multiple tumor-related pathways as cell proliferation, cell cycle, cell proliferation, apoptosis, and metastasis as well as affecting the epigenetic and immune regulation [25]. However, it is believed that more features of *miR-29s* still need to be explored. Moreover, *miR-29s* are frequently down-regulated in multiple cancer types (e.g. NSCLC), while the mechanisms responsible for the dysregulation have not been fully elucidated. In view of this, we here comprehensively explored the function and regulator mechanism of the down-regulated *miR-29s* in NSCLC.

In the present study, *miR-29s* were shown to directly target and down-regulate the expression of SETDB1 expression at the levels of transcription and translation, which finally up-regulated the expression of TP53. Furthermore, as a direct transcriptional regulator of *miR-29s* [19], TP53 was confirmed to up-regulate the expression of *miR-29s*, and thereby down-regulated SETDB1 expression. Therefore, a regulatory circuitry comprising TP53, *miR-29s*, and SETDB1 was suggested in the carcinogenesis of NSCLC. Interestingly, Sun et al. [11] previously observed that SETDB1 and TP53 modulated the expression of each other in NSCLC, but the potential mechanism of this novel interaction was not elucidated. Of note, our newly identified TP53/*miR-29s*/SETDB1 regulatory circuitry partly explains the interaction of TP53 and SETDB1, in which TP53 represses SETDB1 expression via the intermediator *miR-29s*. However, the TP53/SETDB1 feedback loop remains to be further studied. On the other side, the TP53 pathway is inactivated in most human cancers, and restoration of TP53 may provide a new, effective approach for cancer therapy [26]. *miR-29s* have been previously reported to participate in the activation of TP53 pathway [27]. Adding evidence to this regulation, we here identified that *miR-29s* are involved in the restoration of TP53 through directly targetting SETDB1, a negative regulator of TP53 [11]. These facts might suggest the restoration of TP53 by *miR-29s* plays a considerable contribution in *miR-29s* performing their anticancer function.

As a target of TP53 repression [6], SUV39H1 was shown to be indirectly targetted by *miR-29s* in this study. Importantly, SUV39H1 specifically catalyzes the trimethylation of H3K9me3 and governs global H3K9me3 level [6,28], while SETDB1 mainly methylates H3K9 to the di- and trimethyl states (H3K9me2 and H3K9me3) [29]. Currently, H3K9 methylation has emerged as an important modification that is associated with gene silencing and heterochromatin formation [30]. High-resolution mapping of histone modification patterns has shown that H3K9me2 and H3K9me3 are enriched in the transcriptional start sites of silenced genes [31]. Therefore, we proposed that *miR-29s* may play as a regulator of histone H3K9 methylation by targetting directly SETDB1 and indirectly Suv39h1 in NSCLC. The sequential analysis demonstrated that modulation of *miR-29s* expression indeed affected the levels of H3K9me2 and H3K9me3 in our working model, suggesting that dysregulation of *miR-29s* contributes to the aberrant H3K9 methylation status in NSCLC. Moreover, H3K9me3 serves as a diagnostic marker in both recurrence and distant metastasis in lung cancer patients [32]. Previously, *miR-29s* have been proved to effectively regulate DNA methylation through down-regulating the DNA methyltransferases (DNMTs) in NSCLC [15]. Altogether, these data provide functional links between *miR-29s* and epigenetic modulation in NSCLC and show potential insight for an effective therapeutic method to eliminate the aberrant epigenetic modifications (DNA methylation, H3K9 methylation) through the use of synthetic *miR-29* oligonucleotides.

In summary, our findings highlight a TP53/*miR-29s*/SETDB1 regulatory circuitry, whose dysfunction may contribute to tumor survival and progression of NSCLC (Figure 6E). Our study also offers new insight into the biological significance conferred by *miR-29s* in the regulation of H3K9 methylation and provide a potential therapeutic approach in targetting TP53/*miR-29s*/SETDB1 regulatory circuitry for the treatment of NSCLC.

## Abbreviations

ATCC: American Type Culture Collection
DMEM: Dulbecco’s modified Eagle medium
GAPDH: glyceraldehyde 3-phosphate dehydrogenase
H3K9me3: Lys^9^ residue on histone H3
NSCLC: non-small cell lung cancer
qPCR: quantitative real-time PCR
Scr NC: scramble oligonucleotide used as negative control
SETDB1: SET domain, bifurcated 1
TP53: tumor protein 53
